# Ion Chromatography as a Sustainable Alternative for Monitoring Ethanol and Free Glycerol in Biodiesel

**DOI:** 10.1021/acsomega.5c01406

**Published:** 2025-07-10

**Authors:** Ramon S. B. Ferreira, Patrícia T. de Souza, Daniel Gonçalves, Rafaela M. dos Passos, Klicia Araujo Sampaio, Antonio J. A. Meirelles, Eduardo A. Caldas Batista

**Affiliations:** Laboratory of Extraction, Applied Thermodynamics, and Equilibrium (EXTRAE), School of Food Engineering (FEA), 28132 University of Campinas (UNICAMP), 80 Monteiro Lobato St., 13083-062 Campinas, SP, Brazil

## Abstract

The amounts of glycerol and ethanol in purified biodiesel are standardized by regulatory agencies to ensure the performance of the biofuel in combustion. Therefore, the correct measurement of these components is essential to evaluate the final quality of biodiesel and to monitor its synthesis. In addition, evaluation of the composition in ethanol and glycerol is also relevant in modeling the kinetics of the transesterification reaction. Ion chromatography was evaluated as an alternative technique to identify and quantify ethanol and free glycerol in biodiesel samples from heterogeneous, homogeneous, and enzymatic catalysis. The analytical method exhibited detection limits of 0.1 and 0.94 mg·L^–1^ and quantification limits of 0.3 and 2.83 mg·L^–1^ for glycerol and ethanol, respectively. Additionally, the analytical method demonstrated suitable repeatability (RSD_glycerol_ = 0.14% and RSD_ethanol_ = 0.80%), reproducibility (RSD_glycerol_ = 1.27% and RSD_ethanol_ = 3.32%), and selectivity, proving to be reliable in relation to the matrix effects according to the validation guide of the Association of Official Analytical Chemists (AOAC). Ion chromatography has been demonstrated to be an alternative technique that is adequate for the quantification of ethanol and glycerol in different alkyl ester systems. Moreover, the technique dispenses the use of organic solvents, resulting in a considerable reduction of toxicity, analysis time, and analytical costs, and contributes to both environmental and operational securities.

## Graphical abstract

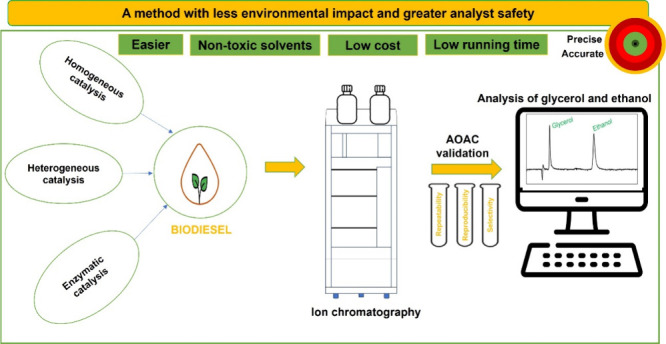

## 1 Introduction

Over the years, biodiesel has been assumed as an alternative and renewable fuel to fossil ones. Biodiesel is a mixture of alkyl esters of fatty acids that are mainly obtained from the transesterification of triacylglycerols from vegetable oils and fats with low-carbon-chain alcohols.^1–3^

Although other alcohols, such as propanol, butanol, and amyl alcohol, can be used in the transesterification process, methanol and ethanol are preferred. The bioethanol (ethanol produced from biomass) can be used in the synthesis of biodiesel and, being a renewable resource, it becomes the best alternative in terms of sustainability, giving an advantage to fully renewable biodiesel.^1,2,4^ Technological and industrial advances aim to improve the efficiency and sustainability of energy production. There are OECD/FAO forecasts that global bioethanol production will increase to 137 billion liters in 2026. Brazil is expected to be the main contributor to this increase, followed by the USA, China, and Thailand.^5^

The transesterification of vegetable, fish, or microbial oils with ethanol by different catalysis for the synthesis of fatty acid ethyl esters (FAEEs - biodiesel) is already widespread in the literature and relates to conditions with yields within 95% and 99%.^6–9^ Besides the alkyl esters, there is the generation of glycerol as a valuable byproduct, which represents about 10% in mass.^10,11^ However, the presence of glycerol is considered a contaminant in biodiesel because it can form deposits in combustion engines. Such a byproduct is obtained either from homogeneous or heterogeneous catalysis.

The most common homogeneous catalysts are acids, as sulfuric or hydrochloric, or alkaline compounds, as sodium hydroxide or potassium hydroxide, the latter being the most used in biodiesel production due to its high conversion rates. On the other hand, biodiesel may contain free glycerol, alcohol, catalyst, salts, soaps, water, free fatty acids, and acylglycerols (mono, di, or triacyclglycerols).^12^ Heterogeneous catalysts are insoluble solids that can be easily removed from the mixture. Anion exchange resin, CaO, MgO, and CaO impregnated with silica and hydrotalcite are examples of these catalysts.^12^ Biodiesel from enzymatic transesterification is obtained using enzymes as free (homogeneous catalysis) or immobilized (heterogeneous catalysis) lipases, for instance. This procedure, besides effluent reduction, generates a higher quality glycerol than those from acid or alkaline procedures,^13^ for instance.

Regardless of the catalytic route, the generated biodiesel needs to go through purification steps in order to meet the regulatory standards, such as high reaction of acylglycerols (>96.5% by mass), low compositions in free glycerol (0.02% by mass), water (<200 mg·kg^–1^), alcohol (<0.2% by mass), salts (<5 mg·kg^–1^), metals (<1 mg·kg^–1^ per element), and free fatty acids (<0.3 mg_KOH_·g^–1^).^14,15^

The presence of free glycerol above the concentration regulated by ABNT NBR, ASTM D, and EN/ISO (0.02% in mass) may generate deposits in the engine and impair its proper operation.^16^ Residual alcohol may also be prejudicial to the biodiesel security because a minimal concentration of alcohol can decrease the biodiesel flash point that must be higher than 100 °C.^17^

Traditionally, free glycerol can be measured by gas chromatography according to the procedure suggested by the American Society for Testing and Materials (ASTM, D 6584-0) or by the European Committee for Standardization (EN 14105). However, alternative methods have been developed for the quantification of free glycerol in biodiesel samples, as the photoenzymatic method,^16^ capillary electrophoresis using multiple short-end injection (CE SE/MI),^18^ high-performance liquid chromatography with a refractive index detector (HPLC - RID),^19^ and an alternative spectrophotometric method based in periodate oxidation of glycerol and formation of formaldehyde, followed by reaction with acetylacetone and spectrophotometric measurement at 410 nm has also been implemented for the determination of free glycerol.^20^

In turn, the alcohol concentration in biodiesel is usually assessed by gas chromatography with a flame ionization detector (GC-FID) and headspace sampling, according to the procedure suggested by the European Committee for Standardization (EN ISO 14110), indicated by standard EN ISO 14214, for the determination of methanol in biodiesel, and by RANP 07/08, according to the procedure suggested by the Resolution of the Agência Nacional do Petróleo, Gás Natural e Biocombustveis, for the determination of both methanol and ethanol. Furthermore, the determination of residual ethanol in biodiesel has also been carried out by ^1^H NMR^17^ or other methods such as HPLC-RID,^21^ or by near-infrared spectroscopy, which requires the use of chemometric tools, in addition to indirect methods such as determining the flash point of biodiesel.^22^ However, some of these methods still exhibit significant limitations for routine use, mainly the need for multiple steps for derivatization and sample preparation, which make the process more labor-intensive and less practical. In addition, many of these methods involve the use of toxic solvents that may pose significant health hazards, such as tetrahydrofuran (THF),^23–25^ commonly used in the quantification of glycerol by HPLC. In the case of NMR spectroscopy, the high operational and maintenance costs represent an additional barrier to its application in routine analyses.

The consolidated methods for quantifying glycerol and ethanol by gas chromatography (EN ISO 14105 and RANP 07/08, respectively) also use high-cost materials, such as fibers and headspace sampling accessories, in addition to using reagents with high toxicity (pyridine, butanetriol, hexane, heptane, *N*-methyl-*N*-(trimethylsilyl)­trifluoroacetamide - MSTFA). Therefore, an option for a methodology that can simultaneously analyze glycerol and ethanol, offering fast and simple sample preparation, analytical robustness, and operational safety, would be more appealing for the quality control and scientific research sectors.

Ion chromatography and liquid–liquid extraction are proposed here as alternative methodologies for the simultaneous evaluation of ethanol and free glycerol in biodiesel samples. Ion chromatography is a technique that allows the development of analytical procedures with simple sample preparation, improved sensibility and analytical selectivity, lower toxicity of reagents, reduced analysis costs (water is used as a partial or complete substitute of organic solvents), and a reduced reagent amount, generating fewer residual effluent volumes that are generally destined to incineration. Then, this analytical procedure may contribute to operational security and environmental preservation.^26,27^

The ion chromatography method was already adopted for the analysis of cations (sodium, potassium, lead, calcium, and magnesium) and anions (acetate, formate, chloride, phosphate, and sulfate) found in biodiesel and exhibited suitable results when compared with conventional analytical methods EN ISO 14538 and EN 16294, EN ISO 20846, EN ISO 20884 and ASTM D5453.^26–32^ Short-chain carboxylic acids, determined as carboxylate anions (formate, acetate, propionate, butyrate, valerate, and hexanoate) were also assessed by ion chromatography which allowed to carry out studies on the kinetics of lipid oxidation and levels of acidity, corrosivity, and oxidation of biodiesels with different fatty acid alkyl ester compositions.^33,34^

Therefore, this study aimed to develop an easy, safe, and ecofriendly analytical procedure for the simultaneous evaluation of ethanol and free glycerol in biodiesel based on the ion chromatography technique. The analytical procedure can be used for product quality assurance as well as the acquisition of transesterification kinetics data.

## 2 Materials and Methods

### 2.1 Materials

Palm olein used in the transesterification procedure was kindly donated by Agropalma S.A. (Limeira, SP, Brazil). The Purolite A503S resin (heterogeneous catalyst) was acquired from Purolite (Brazil). This resin is sold as Cl^–^ and it was activated with NaOH to substitute ions Cl^–^ by OH^–^ before being used in the transesterification catalysis.^35^

Ethanol (analytical purity >99.0%), glacial acetic acid (100% purity), and sodium hydroxide solution (50% by mass, in water) were purchased from Merck (Germany). Sodium ethoxide (21% by mass, in ethanol), sodium acetate (99.9% purity), glycerol (HPLC grade, 99.5% purity), and standard esters ethyl oleate, ethyl linoleate, ethyl palmitate, and ethyl stearate (99% purity) were acquired from Sigma-Aldrich (U.S.A.). Eversa Transform 2.0 free liquid lipase was acquired from Novozymes (Denmark). Ultrapure water was obtained from a Milli-Q device (Merck Milli Pore, Germany).

### 2.2 Methods

#### 2.2.1 Chromatographic Conditions

Samples were analyzed in an ion chromatograph, model 940 Professional IC Vario, equipped with an isocratic pump system, an amperometric detector (working in Pulsed Amperometric Detection - PAD mode, at 45 °C, with Wall-Jet cell containing Au working electrode and Pd reference electrode), a Metrosep Carb 2 -150/4.0 column, and a precolumn with the same internal composition of the main column (Metrohm, Switzerland). The Metrosep Carb 2-150/4.0 IC column is particularly suitable for the determination of carbohydrates using alkaline eluents and PAD. It is stable in the range of pH 0 to 14 and separates mono- and disaccharides, being also suitable for the analysis of sugar alcohols, oligosaccharides, and short-chain alcohols. Operational chromatographic conditions were optimized in terms of: (i) eluent composition by ranging the concentrations of sodium acetate from 10 to 100 mmol·L^–1^ and sodium hydroxide (NaOH) from 100 to 400 mmol·L^–1^; (ii) oven temperature between 35 and 55 °C; and (iii) injection flow rate from 0.6 to 0.8 mL·min^–1^.

The equipment was first equilibrated by pumping the eluent through the injection line and chromatographic column during 20 min to establish the detector signal. After each run, the sampling line (Model 858 Professional Sample Processor, Metrohm, Switzerland) was washed with ultrapure water during 1 min, whereas the injection line was conditioned with the sample for 3 min. An injection volume of 20 μL and a chromatographic run of 10 min were adopted in all analyses.

Analytes were quantified by external standardization with calibration curves constructed for glycerol from 0.1 to 100 mg·L^–1^ and for ethanol from 5 to 100 mg·L^–1^. Chromatograms were then evaluated in terms of analytical selectivity and chromatographic resolution.

#### 2.2.2 Transesterification Reactions

Biodiesel samples were prepared by three different transesterification routes (different catalysts): (i) homogeneous catalysis with sodium ethoxide, (ii) heterogeneous catalysis with Purolite A503S resin, and (iii) enzymatic transesterification with Eversa Transform 2.0 free liquid lipase. Reactions were carried out in a jacketed reactor of 50 mL, as procedure described by Ferreira et al. (2021).^35^ The reactor was placed on a magnetic stirrer (IKA, MAG HS7, U.S.A.) and connected to a thermostatic bath (Paar Physica, Viscotherm VT2, Germany) for temperature control. Catalysts and reagents (palm olein and ethanol) were weighed in an analytical balance (precision of 1 × 10^–4^ g, Precisa, XT 220A, Brazil), and immediately mixed inside the reactor.^35^ Details of each transesterification reaction are described next.

##### 2.2.2.1 Biodiesel from Homogeneous Catalysis (BO)

Reactions were carried out according to Silva et al.^36^ at 60 °C, stirring speed of 400 rpm during 60 min, ethanol to palm olein molar ratio of 16:1, and catalyst (sodium ethoxide) amount of 1% palm olein mass. Acetic acid was added to the mixture to stop the reaction in an equivalent molar proportion of the catalyst.

##### 2.2.2.2 Biodiesel from Heterogeneous Catalysis (BH)

Reactions were carried out according to Ferreira et al.^35^ by assuming a resin mass of 17.6% of palm olein mass, temperature of 49.4 °C, ethanol to palm olein molar ratio of 12.85:1 and stirring speed of 400 rpm during 10 h. The process was stopped by removal of the resin.

##### 2.2.2.3 Biodiesel from Enzymatic Catalysis (BE)

Enzymatic transesterification reactions were done in accordance with procedure reported by Rosset et al.^37^ with modifications, using Eversa Transform 2.0 enzyme, also known as lipase NS 40116. The temperature used was 35 °C, 0.5% lipase, and 15% water, both in relation to a palm olein mass, ethanol to palm olein molar ratio of 4.5:1, and a stirring speed of 400 rpm during 36 h. The experiment was stopped by separating the enzyme by centrifugation (Hettich, 380 R, Germany) at 5 °C.

##### 2.2.2.4 Purified Biodiesel from Homogeneous Catalysis (BP)

The biodiesel obtained from the homogeneous reaction was purified as described below. The purification steps were carried out according to the study by Silva et al.^36^ After the transesterification reaction, the excess ethanol was evaporated under vacuum using a rotary evaporator (Marconi, MA-120, Brazil). Next, the mixture was placed in a separation funnel, and after decantation, the ethyl ester-rich phase was removed, washed, and dried to obtain purified biodiesel.

#### 2.2.3 Sample Preparation and Extraction of Analytes

Ethanol and glycerol recovery from biodiesel from homogeneous catalysis samples (BO) was evaluated aiming also to optimize the procedure of sample preparation. For the study of extraction conditions, 15 min of extraction was defined in preliminary tests (Figure S1 of the Supporting Information). For the recovery trials, temperatures of 30, 45, and 60 °C were evaluated with stirring speed kept constant at 500 rpm. After that, the effect of 200, 500, and 800 rpm stirring speeds was evaluated at the optimal temperature.

For the extraction of analytes, about 0.1 mL of biodiesel sample was weighed on an analytical balance (precision of 1 × 10^–4^ g, Precisa, XT 220A, Brazil), and 10 mL of ultrapure water was added into a 15 mL tube. Tubes were then simultaneously shaken and heated for 15 min inside a dry bath (Kasvi, K80-200, Brazil) under optimal conditions (section 3.2). After that, tubes were kept at rest for 20 min under room conditions for phase separation before sampling; 250 μL of aqueous phase was then collected and diluted in 9.75 mL of ultrapure water. The solution was filtered in a syringe filter (hydrophobic PTFE filter, 0.45 μm porosity, 25 mm diameter) and placed inside an ion chromatograph vial (polypropylene vial of 11 mL) for analysis.

#### 2.2.4 Method Validation

The chromatographic method was validated by following the Association of Official Analytical Chemists (AOAC) guide.^38^ Analytical curves were appraised in terms of linearity, determination coefficient (*R* ^2^), repeatability, and reproducibility.

For analyte recovery, different contents of ethanol and glycerol, both in HPLC grade, were added to biodiesel samples obtained from homogeneous catalysis (BO), heterogeneous catalysis (BH), enzymatic catalysis (BE), and purified biodiesel (BP) in the optimal conditions of sample preparation and analyte extraction (section 2.2.3).

The matrix effects were assessed by using the standard esters ethyl oleate (48.10 wt %), ethyl linoleate (9.92 wt %), ethyl palmitate (37.74 wt %), and ethyl stearate (4.24 wt %) in a mixture to experimentally represent a biodiesel with a fatty composition similar to that from palm olein. Ethanol and glycerol in HPLC grade were added to the model biodiesel mixture, both in different compositions within the analytical curve range (0.0, 5.0, 25.0, 50.0, and 100.0 mg·kg^–1^ of ethanol, and 0.1, 1.0, 25.0, 50.0, and 100.0 mg·kg^–1^ of glycerol). Moreover, the suppression or addition of analyte signals was also evaluated by the matrix factor as described by Viswanatha et al. (2007).^39^ The matrix factor (MF) was calculated by the relation between the chromatographic peak response in the presence of matrix (PM) and the peak response in the absence of matrix (AM):

MF=PM/AM 1

The detection (LOD) and quantification (LOQ) limits were also assessed according to the AOAC official guide:^38^

LOD=3.3×s/S 2

LOQ=10×s/S 3

where *s* is the standard deviation of the linear coefficient and *S* is the arithmetic mean of the angular coefficients of the analytical curves.

#### 2.2.5 Stability Test

Short-term stability tests were performed for the analytes of interest (ethanol and glycerol) in three weeks. After synthesis, biodiesel samples (BO, BH, and BE) were analyzed by the proposed chromatographic method and then stored at −20 °C. For the sequential analysis after one week, biodiesel samples were defrosted at room temperature (∼25 °C) and vigorously shaken before the analyte extraction. All samples were analyzed and quantified by the calibration curves prepared for ethanol and glycerol. This study was carried out to verify the influence of biodiesel storage on the quantification of analytes because samples are not always analyzed in the sequence in which they are produced.

## 3 Results and Discussion

### 3.1 Optimization of Chromatographic Conditions

Separation of analytes was optimized by assuming different eluent flow rates and compositions, besides the oven temperature (Table 1). Analytical selectivity was evaluated by the resolution of chromatographic peaks, assuming chromatograms obtained for 50 mg·kg^–1^ of ethanol and 1 mg·kg^–1^ of glycerol (chromatographic profile like in Figure 3A), according to the procedure described by the AOAC official guide.^38^

**Table 1:** Analytical Selectivity (for 50 mg·kg^–1^ of Ethanol and 1 mg·kg^–1^ of Glycerol) at Different Concentrations of Eluent Composition, Flow Rate, and Oven Temperature

	eluent composition (mmol·L^–1^)						
fixed condition	sodium acetate	sodium hydroxide	*t*_1_	*t*_2_	*w*_1_	*w*_2_	Rs^a^
eluent flow: 0.8 mL·min^–1^; oven temperature: 45 °C	10	100	2.21	5.94	0.2	0.3	14.92
	50	200	2.09	6.08	0.15	0.3	17.73
	100	400	2.05	6.46	0.15	0.35	17.64
fixed condition	oven temperature (°C)						
eluent composition: 50 mmol·L^–1^ sodium acetate and 200 mmol·L^–1^ NaOH; eluent flow: 0.8 mL·min^–1^	35		2.12	6.49	0.2	0.4	14.57
	45		2.09	6.08	0.15	0.3	17.73
	55		2.06	5.71	0.2	0.35	13.27
fixed condition	eluent flow (mL·min^–1^)						
oven temperature: 45 °C; eluent composition: 50 mmol·L^–1^ sodium acetate and 200 mmol·L^–1^ NaOH	0.6		2.77	8.01	0.25	0.45	14.97
	0.8		2.09	6.08	0.15	0.3	17.73
	1.0		1.69	4.59	0.2	0.3	11.6

a Peak resolution, Rs = 2 (*t*_2_ – *t*_1_)/(*w*_1_ + *w*_2_); *t*_1_ and *t*_2_ are retention times of glycerol and ethanol (min), respectively; *w*_1_ and *w*_2_ are widths at half peak height of glycerol and ethanol, respectively.

Glycerol was eluted in a shorter time using an eluent with a higher concentration of sodium acetate and sodium hydroxide, whereas ethanol exhibited an opposing behavior. An eluent flow rate of 0.8 mL·min^–1^ and an oven temperature of 45 °C were adopted for the evaluation of the eluent composition effect on the elution of analytes.

The Rs values calculated for the three compositions were higher (Table 1) than the values recommended by the AOAC guide (Rs ≥ 1.0) and Food and Drug Administration (FDA, Rs ≥ 2.0). However, an eluent with 50 mmol·L^–1^ sodium acetate and 200 mmol·L^–1^ sodium hydroxide was chosen because it resulted in a Rs value slightly higher than those of the other compositions (Table 1).

For the appraisal of the oven temperature effect, an eluent composition of 50 mmol·L^–1^ sodium acetate and 200 mmol·L^–1^ and flow rate of 0.8 mL·min^–1^ were assumed, where the higher Rs value was calculated for 45 °C. Finally, the eluent flow was evaluated assuming the optimal oven temperature (45 °C) and composition (50 mmol·L^–1^ sodium acetate and 200 mmol·L^–1^ sodium hydroxide). The best result was reached for an eluent flow rate of 0.8 mL·min^–1^.

Therefore, the optimum chromatographic conditions were eluent with 50 mmol·L^–1^ sodium acetate and 200 mmol·L^–1^ sodium hydroxide, an oven temperature of 45 °C, a flow rate of 0.8 mL·min^–1^, and a running time of 10 min.

### 3.2 Extraction of Analytes from Biodiesel Samples

The extraction conditions of the analytes glycerol and ethanol were studied by evaluating the effect of the temperature (Figure 1) and stirring speed (Figure 2). Based on preliminary studies, the extraction time was fixed at 15 min (please, see Figure S1 in the Supporting Information). The optimum temperature of 45 °C was attained based on the maximum extraction of glycerol and ethanol.

**Figure 1:**
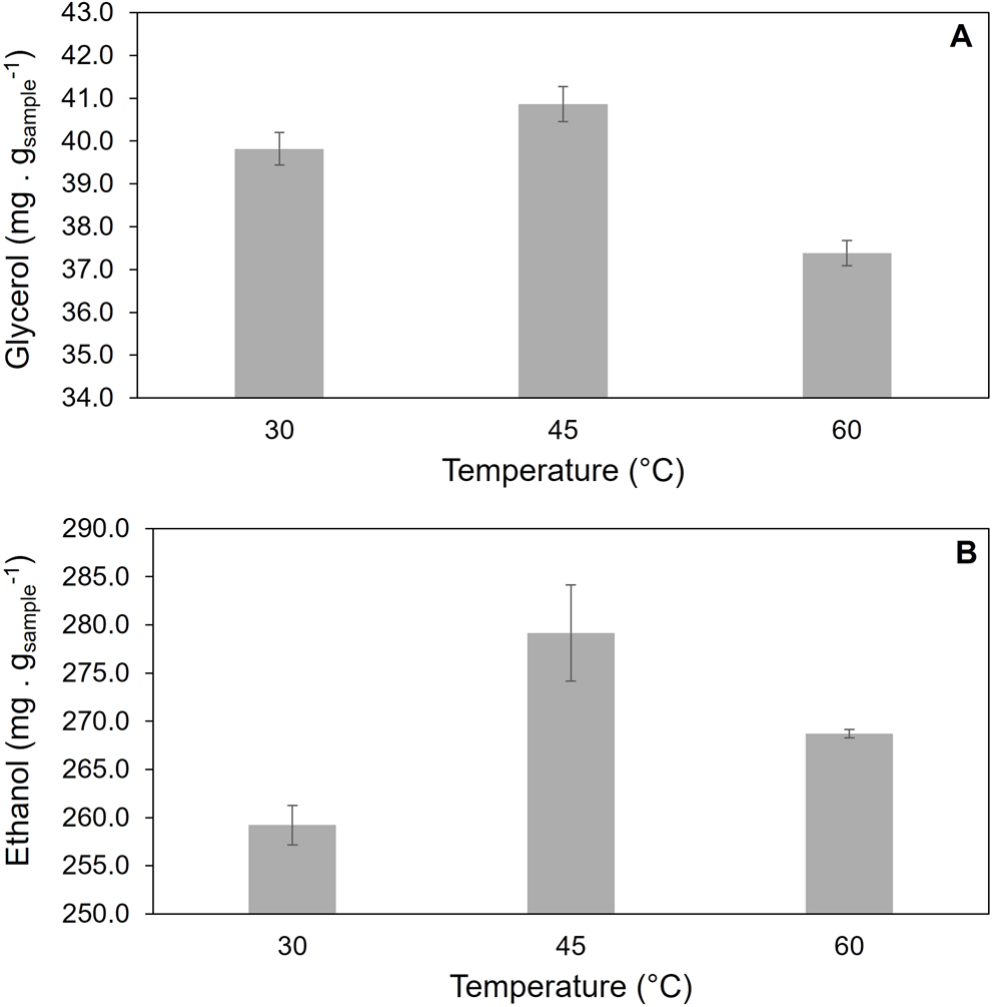
Evaluation of the extraction temperature of (A) glycerol and (B) ethanol from biodiesel obtained by homogeneous catalysis (BO) at 500 rpm and 15 min of extraction time.

**Figure 2:**
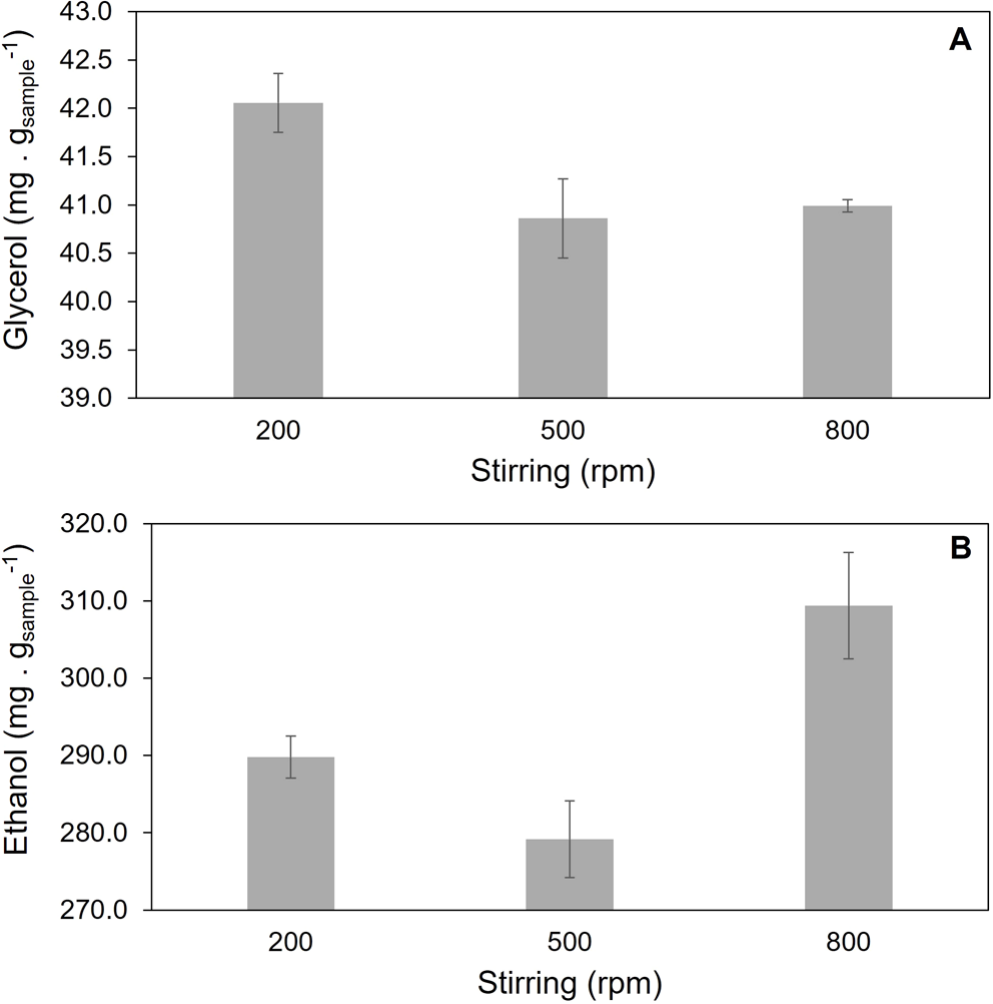
Evaluation of stirring speed in the extraction of (A) glycerol and (B) ethanol from biodiesel obtained by homogeneous catalysis (BO) keeping the temperature fixed at 45 °C, and 15 min of extraction time.

Because a small difference in glycerol extraction was observed between 500 and 800 rpm (40.86 and 40.99 mg·g_sample_^–1^, respectively), the stirring speed of 800 rpm was chosen based on the maximum ethanol recovery (309.37 mg·g_sample_^–1^), which showed a pronounced difference compared to 200 and 500 rpm (289.77 and 279.15 mg·g_sample_^–1^, respectively). Therefore, the optimum extraction conditions were as follows: a stirring speed of 800 rpm, a temperature of 45 °C, and an extraction time of 15 min.

### 3.3 Parameter Validation

#### 3.3.1 Analytical Curve Linearity

Analytical curves were constructed for glycerol and ethanol by relating the chromatographic peak area to the analyte concentration (mg·L^–1^). According to Table 2, both curves exhibited good linearity (R^2^ > 0.99) agreeing with the AOAC official guide.^38^

**Table 2:** Analytical Parameters for Glycerol and Ethanol are from Analytical Curves and Chromatogram Data

component	retention time (min)^a^	angular coefficient^b^	linear coefficient	SD^c^	*R* ^2^	LOD (mg·L^–1^)	LOQ (mg·L^–1^)	linear range (mg·L^–1^)
glycerol	2.09	112.78	17.92	3.42	>0.99	0.10	0.30	0.1–100
ethanol	6.08	3.05	6.95	0.86	>0.99	0.94	2.83	5–100

a In the chromatographic run.

b Arithmetic mean of the angular coefficients of the curves obtained during the study.

c Standard deviation of the linear coefficients.

The analytical parameters of glycerol and ethanol, such as retention times, angular and linear coefficients of analytical curves, deviation in the linear coefficient, LOD and LOQ, and concentration ranges of the analytical curves, are available in Table 2. LOQ values for both analytes are below those required by the regulatory fuel agencies, which permit a free glycerol amount of 0.02% in mass (or 200 mg·kg^–1^) and a residual ethanol content of 0.2% in mass (or 2000 mg·kg^–1^) in the purified biodiesel,^15,38,40^ even considering a 4,000-fold dilution in the sample preparation. This demonstrates that the analytical method has an adequate sensibility for the purpose of its application.

Chromatograms for samples containing 1 and 60 mg·L^–1^ glycerol and 10, 50, and 60 mg·L^–1^ ethanol are shown in Figure 3. The signal intensities are close for concentrations of 1 mg·L^–1^ glycerol and 50 mg·L^–1^ ethanol (Figure 3A), but they are different considering the lowest (1 mg·L^–1^ glycerol and 10 mg·L^–1^ ethanol, Figure 3B) or the highest concentrations (60 mg·L^–1^ glycerol and 60 mg·L^–1^ ethanol, Figure 3C). The glycerol signal is more prominent than the ethanol one because as the glycerol concentration increases, its signal becomes much higher than the ethanol one (Figure 3).

**Figure 3:**
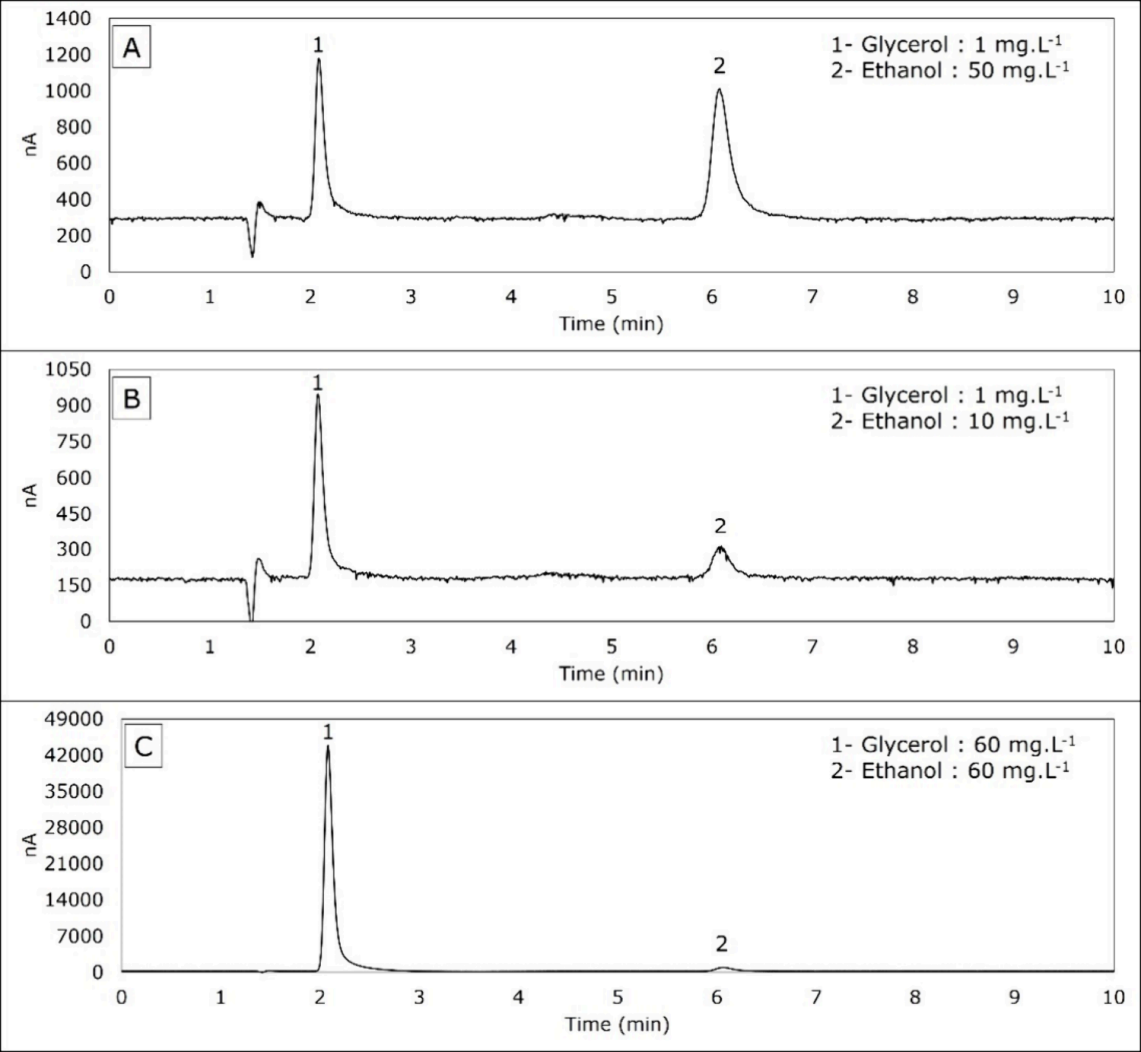
Chromatograms for different concentrations of glycerol and ethanol. (A) 1 mg·L^–1^ glycerol and 50 mg·L^–1^ ethanol, (B) 1 mg·L^–1^ glycerol and 10 mg·L^–1^ ethanol, and (C) 60 mg·L^–1^ glycerol and 60 mg·L^–1^ ethanol.

#### 3.3.2 Repeatability and Reproducibility

For the tests of repeatability (three times on the same day) and reproducibility (five different days in three different weeks, resulting in 15 samples), outcomes were expressed as the relative standard deviation (RSD, Table 3) according to the AOAC official guide.^38^

**Table 3:** Relative Standard Deviation (RSD) for Repeatability and Reproducibility Tests

compound	concentration (mg·L^–1^)^a^	intraday^a^	concentration (mg·L^–1^)^b^	interday^b^
glycerol	36.51 ± 0.05	0.14	36.94 ± 0.47	1.27
ethanol	31.67 ± 0.25	0.80	31.94 ± 1.06	3.32

a On the same day.

b In different days.

Samples were prepared from ethanol and glycerol standards at a concentration of 35 mg·L^–1^ for both analytes and analyzed at three different times (on the same day and on different days in different weeks). RSD of the repeatability test was lower than 1%, which is much lower than the value recommended by the AOAC official guide (RSD < 6% for 10 mg·L^–1^ of analyte).^38^ For the reproducibility test, RSD < 11% (considering 10 mg·L^–1^ of analyte) is required.^38^ Results are in accordance since RSD values for reproducibility for different days and weeks were lower than 5%. This means that the proposed analytical method is indeed adequate to assess ethanol and glycerol in biodiesel with good repeatability and reproducibility.

#### 3.3.3 Analyte Recovery or Accuracy

Recovery data for ethanol and glycerol are presented in Tables 4 and 5, respectively. To analyze the analyte recovery, ethanol and glycerol, both HPLC grade, were added to biodiesel samples obtained from homogeneous catalysis (BO), heterogeneous catalysis (BH), enzymatic catalysis (BE), and purified biodiesel (BP). Initial, added, and total compositions of analytes in the samples are also summarized in Tables 4 and 5. Initial and total concentrations were quantified by ion chromatography.

**Table 4:** Recovery Data for Glycerol

sample^a^	initial^b^ (mg·L^–1^)	added (mg·L^–1^)	total^b^ (mg·L^–1^)	recovery (%)^c^	recovery limits (%)^d^
BO	0.85	0.55	1.68	120.00	75–120
	4.66	5.55	9.42	92.26	75–120
	43.44	55.45	94.95	96.01	85–100
BH	0.82	0.55	1.42	103.65	75–120
	3.73	5.55	8.97	96.66	75–120
	35.26	55.45	94.25	103.90	85–100
BE	11.72	1.04	13.95	109.33	80–115
	11.72	10.68	21.81	97.37	80–115
	10.04	50.7	60.78	100.07	80–115
BP	0.49	0.11	0.63	105.00	75–120
	0.67	1.11	1.81	101.68	75–120
	1.09	5.55	6.28	94.58	75–120

a BO: biodiesel from homogeneous catalysis; BH: biodiesel from heterogeneous catalysis; BE: biodiesel from enzymatic catalysis; BP: purified biodiesel.

b Initial and total concentrations were quantified by ion chromatography.

c Recovery (%) = 100 × Total/(Initial + Added).

d According to the AOAC official guide.^38^

**Table 5:** Recovery Data for Ethanol

sample^a^	initial^b^ (mg·L^–1^)	added (mg·L^–1^)	total^b^ (mg·L^–1^)	recovery (%)^c^	recovery limits (%)^d^
BO	7.31	6.01	10.91	81.91	80–115
	12.94	12.02	21.67	86.82	80–115
	44.32	45.06	86.65	96.95	80–115
BH	6.66	5.61	12.61	102.77	80–115
	12.68	11.22	21.19	88.66	80–115
	43.27	50.47	94.99	101.33	80–115
BE	20.12	5.39	26.52	103.96	80–115
	17.4	10.82	28.64	101.49	80–115
	20.12	50.48	76.27	108.03	80–115
BP	0	5.01	4.81	96.01	75–120
	0	10.01	10.49	104.79	80–115
	0	50.07	53.86	107.57	80–115

a BO: biodiesel from homogeneous catalysis; BH: biodiesel from heterogeneous catalysis; BE: biodiesel from enzymatic catalysis; BP: purified biodiesel.

b Initial and total concentrations were quantified by ion chromatography.

c Recovery (%) = 100 × Total/(Initial + Added).

d According to the AOAC official guide.^38^

Recovery results were evaluated in three concentration ranges for both analytes (ethanol and glycerol). The AOAC official guide^38^ specifies recovery limits according to the magnitude order. For concentrations on the order of 1 ppm, the range is 75–120% recovery, while for concentrations on the order of 10 ppm, the range is 80–115%. Recovery results ranged within 92.26–120.0% for glycerol and within 81.91–108.03% for ethanol. The worst recovery values (120.0% glycerol and 81.91% ethanol) were attained for samples with the lowest analyte concentrations in BO, which can be due to the presence of signal noise or more impurities in samples [2]. However, all results are in accordance with the requirements of the AOAC official guide^38^ and indicate that the procedure adopted for extraction of these analytes is efficient, and the chromatographic method exhibits proper sensibility, selectivity, precision, and accuracy to assess ethanol and glycerol in samples of biodiesel from different catalytic routes. Additionally, the analytical method can also be used for transesterification kinetics studies.

#### 3.3.4 Matrix Effects

The effects of the matrix on analytes were evaluated. The analytical curves at concentrations of 5.0, 25.0, 50.0, and 100.0 mg·L^–1^ of ethanol, and 0.1, 1.0, 25.0, 50.0, and 100.0 mg·L^–1^ of glycerol are represented by solid lines in Figure 4 and identified as standard lines. The curves constructed with the mixture of standard ethyl esters added with ethanol and glycerol at the same concentration for the standard lines are represented by the dotted lines in Figure 4 and identified as matrix lines. It can be observed in Figure 4 that the standard curves practically overlap the matrix curves, indicating the absence of matrix effect and being in accordance with the requirements of the validation official guide of the Association of Official Analytical Chemists (AOAC).

**Figure 4:**
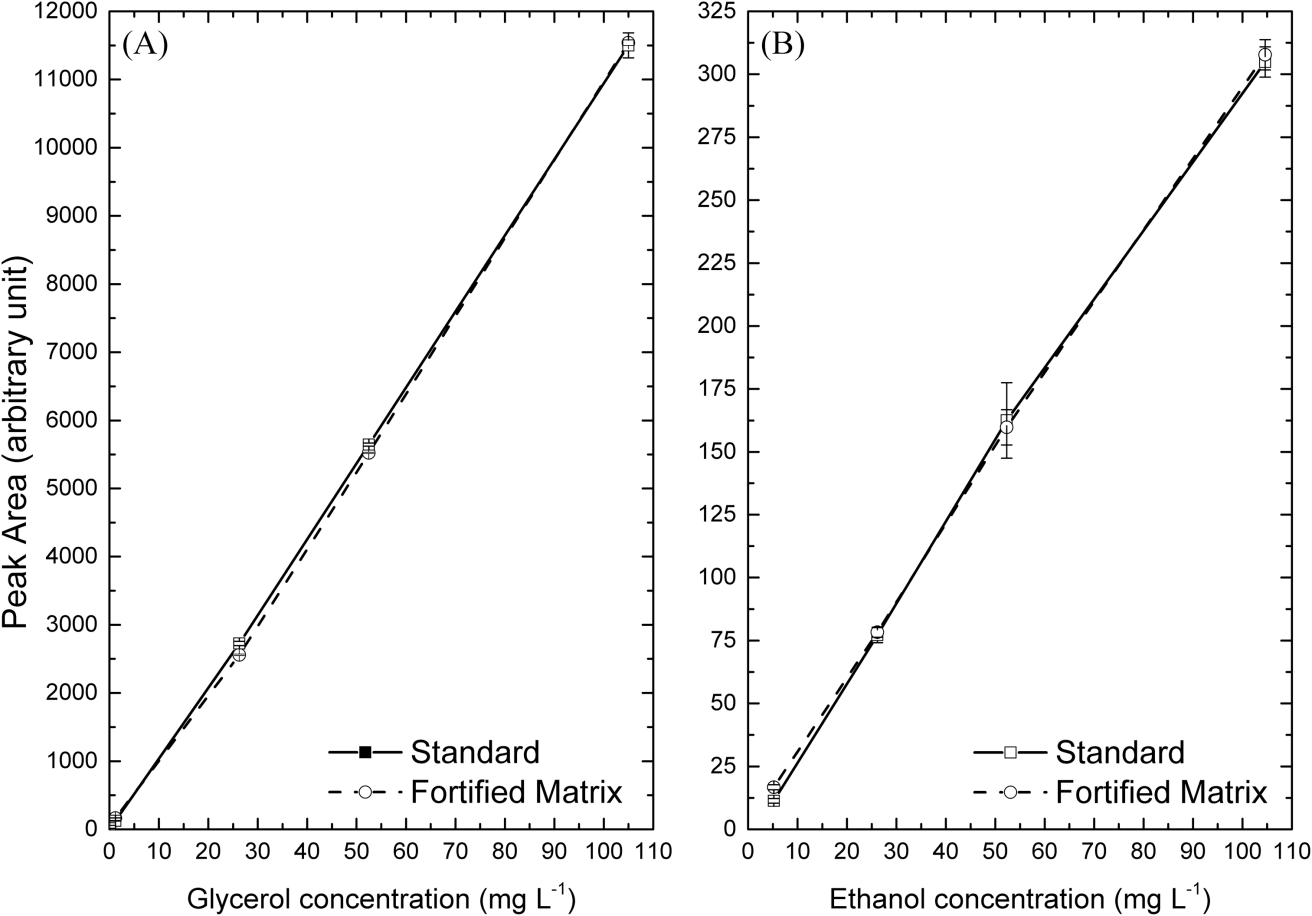
Study curves of matrix effects on analytes: (A) glycerol and (B) ethanol.

The influence of the matrix on the analytes according to the study by Viswanatha et al.^39^ is described in the data from Table 6.

**Table 6:** Concentrations and Average Area of Standards and Fortified Matrices, Matrix Factor (MF), and Relative Standard Deviation (RSD)

		average area of glycerol (arbitrary unit)					average area of ethanol (arbitrary unit)			
test	concentration (mg·L^–1^)	standard	matrix	MF	RSD (%)	concentration (mg·L^–1^)	standard	matrix	MF	RSD (%)
1	105.00	11,500 ± 183	11,538 ± 41	1.00 ± 0.02	1.82	104.60	305 ± 6	307.73 ± 6	1.01 ± 0.04	4.08
2	52.50	5,646 ± 24	5,522 ± 9	0.98 ± 0.01	0.65	52.30	163 ± 15	160 ± 7	0.99 ± 0.07	7.35
3	26.25	2,726 ± 31	2,558 ± 12	0.94 ± 0.01	1.60	26.15	77 ± 3	78 ± 2	1.02 ± 0.02	1.93
4	1.23	126 ± 3	166 ± 4	1.32 ± 0.07	5.05	5.23	11.3 ± 0.9	17 ± 1	1.5 ± 0.1	9.26
5	0.12	14 ± 2	56 ± 2	4.2 ± 0.4	10.4					

A matrix effect was observed for lower concentrations of glycerol (tests 4 and 5, Table 6) since MF values were more than 1. For ethanol, a matrix effect was observed at a low concentration of the analyte (test 4, Table 6) because the MF value was more than 1. Slight or even no influence of the matrix was verified for higher glycerol or ethanol concentrations because MF values are close to 1. According to Viswanatha et al.,^39^ this behavior can be related to favoring or not the analyte availability. In these specific cases, oxidation or reduction reactions of other components of the sample, which may have been extracted together with the analytes, may occur and cause other signals that are detected by the amperometric detector and added to the analyte signals.

Also, as described by Viswanatha et al.,^39^ the MF value close to 1 is not crucial for a reliable method, but RSD values must be less than 15%. All RSD values were less than 15%, independent of the analyte and its concentration (Table 6), meaning that the method is indeed reliable in relation to the matrix effects.

#### 3.3.5 Sample Stability

Stability data for glycerol and ethanol for different biodiesel samples (BO, BH, and BE) are summarized in Table 7.

**Table 7:** Concentration (mg·g_sample_^–1^), Average Concentration, and Relative Standard Deviation (RSD) of Glycerol and Ethanol in Biodiesel Samples Stored at −20 °C for Three Weeks^a^

	concentration (mg·g_sample_^–1^)		
glycerol	week 1	week 2	week 3
BO	53.9 ± 0.3^b^	53.0 ± 0.3^c^	56.4 ± 0.3^a^
BH	24.01 ± 0.05^a^	21.6 ± 0.2^b^	24.2 ± 0.5^a^
BE	60 ± 2^a,b^	55.7 ± 0.4^b^	64 ± 4^a^

ethanol	week 1	week 2	week 3
BO	354 ± 7^c^	400 ± 6^a^	377 ± 12^b^
BH	297 ± 15^c^	345 ± 3^a^	318 ± 2^b^
BE	102 ± 2^a^	108 ± 6^a^	87 ± 13^a^

a BO: biodiesel from homogeneous catalysis; BH: biodiesel from heterogeneous catalysis; BE: biodiesel from enzymatic catalysis. Numbers followed by the same letter in the same line have no statistical difference by the Tukey test (α ≤ 0.05).

Variations in concentrations of glycerol and ethanol throughout the storage period may be associated with analytical errors or random errors. Therefore, overall samples exhibited adequate stability under the evaluated storage conditions besides the fact that the analysis showed suitable reproducibility.

## 4 Conclusions

The extraction of analytes proved to be a robust and reproducible procedure for recovering glycerol and ethanol from different biodiesel samples, allowing their identification and quantification in an aqueous extract with an ion chromatography apparatus.

The proposed chromatographic method exhibited adequate precision, accuracy, repeatability, reproducibility, resolution, and selectivity, being in accordance with the requirements of the AOAC validation official guide. The method showed analytical sensibility to detect and quantify ethanol and glycerol in concentrations lower than the maximum limit required by the regulatory agencies of U.S.A., Europe, and Brazil, and it did not show a matrix effect when considering a mixture of ethyl esters. The analytical methodology, including the extraction of the analytes, provides a considerable decrease in toxicity and costs, in addition to greater environmental and analyst safety, since only water is required as a diluent. Furthermore, due to the low eluent flow rate of 0.8 mL·min^–1^ and the run time of 10 min, the analytical method generates a low residual effluent volume.

Results revealed that the proposed analytical methodology is adequate and can be alternatively adopted for the quantification of glycerol and ethanol in different ethylic esters, including samples from transesterification kinetics studies.

## Supplementary Material



## Notes

Analysis of the time for maximum extraction of glycerol and ethanol from biodiesel samples (PDF)

## †

Imperatriz Sciences Center, Federal University of Maranhão, 100 Principal Avenue, 65915-240 Imperatriz, MA, Brazil

## ‡

Laboratory of Food Technology (LTA), Universidade Estadual do Norte Fluminense Darcy Ribeiro (UENF), 2000 Alberto Lamego Ave., 28013-602 Campos dos Goytacazes, RJ, Brazil.
