# Supplementary material for: Ion Chromatography as a Sustainable Alternative for Monitoring Ethanol and Free Glycerol in Biodiesel
Source: ACS Omega. 2025 Jul 10;10(28):30222–31. doi: 10.1021/acsomega.5c01406 (PMC12290957; doi:10.1021/acsomega.5c01406)
Supplement: Supplementary file 1 [file ao5c01406_si_001.pdf]

Ion Chromatography as a Sustainable Alternative for Monitoring Ethanol and Free Glycerol in Biodiesel

Ramon S. B. Ferreira [a,#*], Patrícia T. de Souza [a], Daniel Gonçalves [a,@], Rafaela M. dos Passos [a], Klicia Araujo Sampaio [a], Antonio J. A. Meirelles [a], Eduardo A. C. Batista [a*]

[a] Laboratory of Extraction, Applied Thermodynamics, and Equilibrium (EXTRAE), School of Food Engineering (FEA), University of Campinas (UNICAMP), 80 Monteiro Lobato St., 13083-062 Campinas, SP, Brazil

*Email: eacbat@unicamp.br

*Email: barros.ramon@ufma.br

FIGURE

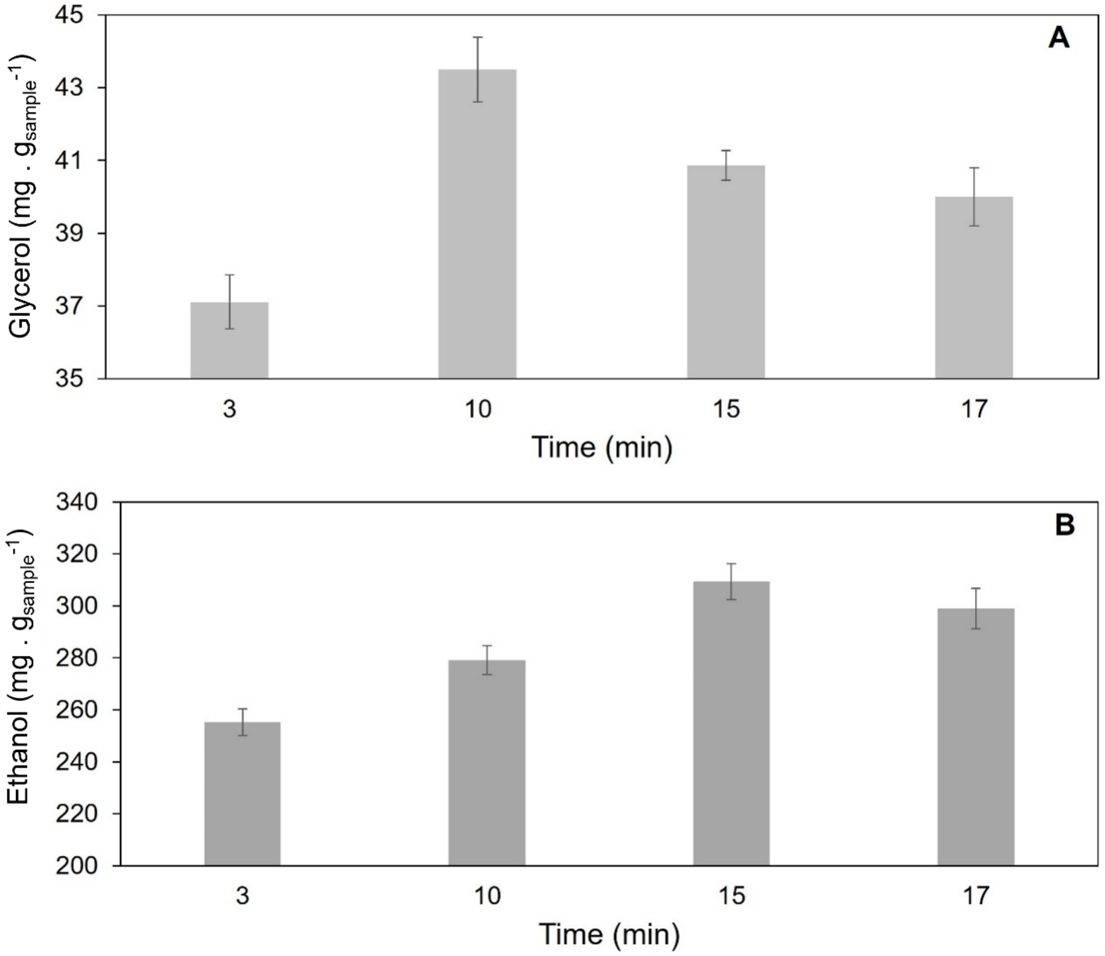

Figure S1. Evaluation of the extraction time for (A) glycerol and (B) ethanol

The time of 15 min was defined in preliminary tests of short kinetics during 17 min, at 45 °C, and under 500 rpm agitation. For glycerol (Fig. S1A), the results ranged from 37.11 to 43.5 $mg \cdot g_{sample}^{-1}$, with a maximum extraction at 10 min. For ethanol (Fig. S1B), the results ranged from 255.31 to 309.37 $mg \cdot g_{sample}^{-1}$, with a maximum extraction at 15 min. The time of 15 min was chosen for the continuation of studies due to the maximum recovery for ethanol at this time and the small difference between the values observed for glycerol at 10 and 15 min, which were 43.5 and 41.0 $mg \cdot g_{sample}^{-1}$, respectively.
